# Interferon-lambda 3 and 4 Polymorphisms Increase Sustained Virological Responses and Regulate Innate Immunity in Antiviral Therapy With Pegylated Interferon-Alpha

**DOI:** 10.3389/fcimb.2021.656393

**Published:** 2021-07-07

**Authors:** Andréa Marques Vieira da Silva, Lucia Elena Alvarado-Arnez, Tamiris Azamor, Leonardo Ribeiro Batista-Silva, Thyago Leal-Calvo, Ohanna Cavalcanti de Lima Bezerra, Marcelo Ribeiro-Alves, Fernanda de Souza Gomes Kehdy, Patrícia Cristina da Costa Neves, Camilla Bayma, Jane da Silva, Alessandro Fonseca de Souza, Marcelo Muller, Elisabete Ferreira de Andrade, Ana Carolina Magalhães Andrade, Eliane Matos dos Santos, Janaína Reis Xavier, Maria De Lourdes De Sousa Maia, Rolando Páez Meireles, Hugo Nodarse Cuni, Guilherme Becker Sander, Paulo Dornelles Picon, Denise C S Matos, Milton Ozório Moraes

**Affiliations:** ^1^ Departamento de Desenvolvimento Tecnológico, Instituto de Tecnologia em Imunobiológico Bio-Manguinhos, Fundação Oswaldo Cruz (FIOCRUZ), Rio de Janeiro, Brazil; ^2^ Coordinación Nacional de Investigación, Universidad Franz Tamayo (UNIFRAZ), Cochabamba, Bolivia; ^3^ Departamento de Hanseníase, Instituto Oswaldo Cruz, Fundação Oswaldo Cruz (FIOCRUZ), Rio de Janeiro, Brazil; ^4^ Laboratório de Pesquisa Clínica em DST/AIDS, Instituto Nacional de Infectologia Evandro Chagas, Fundação Oswaldo Cruz (FIOCRUZ), Rio de Janeiro, Brazil; ^5^ Assessoria Clinica, Instituto de Tecnologia em Imunobiológico Bio-Manguinhos, Fundação Oswaldo Cruz (FIOCRUZ), Rio de Janeiro, Brazil; ^6^ Centro de Investigaciones Clínicas, Centro de Ingeniería Genética y Biotecnologia de Cuba, Havana, Cuba; ^7^ Universidade Federal do Rio Grande do Sul, Hospital de Clínicas de Porto Alegre (HCPA), Porto Alegre, Brazil

**Keywords:** Interferon lambda 3 e 4, hepatitis C, pegylated interferon, sustained virologic response, immune response

## Abstract

Sustained virologic response (SVR) in chronic hepatitis C (CHC) treatment denotes that the host genetics controls the immune response and unequivocally contribute to viral clearance or disease severity. In this context, single nucleotide polymorphisms (SNPs) in the locus of interferon lambda 3 and 4 genes (*IFNL3/4*) have been important genetic markers of responsiveness to CHC as prognostic markers for the pegylated-Interferon-alpha/ribavirin (Peg-IFN-α/RBV). Here, we analyzed 12 SNPs at the *IFNL3/4* region in 740 treatment-naïve patients with CHC infected with hepatitis C virus (HCV) genotypes 1, 2, or 3 treated with Peg-IFN-α/RBV. Individually, rs12979860-CC, rs8109886-CC, or rs8099917-TT were predictive markers of SVR, while rs12979860-CC demonstrated the stronger effect. Besides, the genotypic combination of these three predictors’ genotypes, CC/CC/TT, increased the rate of SVR. Serum levels of cytokines and gene expression analysis on the genes *IFNL3*, *IFNL4*, *IFNA1*, and some of the IFN-stimulated genes (ISGs) were measured in a subgroup of 24 treated patients and 24 healthy volunteers. An antagonist effect was highlighted between the expression of *IFNL3/4* and *IFNA1* mRNA among patients. Besides, a prominent production of the pro-inflammatory chemokines CCL4 and CXCL10 was observed at a 12-week treatment follow-up. Lower serum levels of these chemokines were detected in patients with an rs12979860-CC genotype associated with the better treatment outcome. Also, lower expression levels of the *IFI6*, *IFI16*, *IRF9* genes were observed among rs12979860-CC individuals. In conclusion, a combination of the genotypes at the *IFNL3/4* locus can act as a better marker for the prognosis for virological responses in an admixed Brazilian population presenting the modulating effect over innate immunity and inflammation that are controlling the outcome of the viral infection, but also other infectious diseases. This study is registered on the ClinicalTrials.gov platform (accession number NCT01889849 and NCT01623336).

## Introduction

Chronic hepatitis C (CHC) is the major cause of liver disease, liver cirrhosis, and hepatocellular carcinoma (HCC). There are more than 71 million chronic hepatitis C virus (HCV) carriers worldwide and approximately 399,000 deaths per year (WHO, 2019). Currently, treatments based on direct-acting-antiviral (DAA) are associated with over 90% rates of sustained virologic response (SVR), fewer side effects, outpatient and shorter treatment schemes, and improved adherence compared to interferon-based therapy (Younossi et al., 2017; Ministério da Saúde, 2019). DAA has been introduced as a new public policy to all patients fitting the criteria in Brazilian universal health system.

Nevertheless, the polymorphisms in the *IFNL* genomic region have an influence not only on the treatment outcome but also on viral clearance (Thomas et al., 2009; Balagopal et al., 2010). These polymorphisms in the vicinity of IFN-λ3 are associated with the antiviral activity to another member of the Flaviviridae family helping to understand the immune response of other viruses, pregnancy, and immunosenescence (Bayer et al., 2016; Price et al., 2016; Hemann et al., 2017; Jagger et al., 2017; Rugwizangoga et al., 2019). Two *IFNL3* SNPs, rs12979860 and rs8099917, have a strong association with SVR, mainly in viral genotype 1 HCV-infected patients (Tanaka et al., 2009; Thomas et al., 2009). Host CC genotype at SNP rs12979860 manifests a phenotypic profile related to the spontaneous elimination of HCV and with twice the SVR compared to TT genotype patients treated with Peg-IFN-α/RBV (Ge et al., 2009). Moreover, the predictive value of *IFNL* genotypes is not only limited to the regimen of Peg-IFN-α+RBV therapy. The clinical trial with DAAs therapy combined with IFN-α – SPRINT-2 and ADVANCE trials – showed that rs12979860 affected the treatment outcome, and patients with CC genotype displayed 80% and 90% SVR in the respective trials (Matsuura et al., 2014). Also, an IFN-free INFORM study of mericitabine as a monotherapy, or in combination with danoprevir, suggested that the *IFNL3* genotypes may also predict viral kinetics (Chu et al., 2012; Ramamurthy et al., 2018; Salum et al., 2020).

The frequency of the genotype rs12979860-CC in the United States is higher in Caucasians and Hispanics compared to African-Americans (Li et al., 2011). This group of African ancestry exhibits a lower rate of SVR. The admixed Brazilian population (Manta et al., 2013) might present distinct patterns of linkage disequilibrium (LD) that could impact the SVR. The LD is the nonrandom association of alleles at different loci and might vary between population due to selective forces and population demography (Slatkin, 2008). Thus, the study of *IFNL3/4* genetic variants can have clarity whether the classical SNPs of SVR may be used to predict the HCV treatment outcomes or could also contribute to viral clearance in other infections.

Here, we confirm that SNPs rs12979860-CC, rs8109886-CC, and rs8099917-TT are good prognostic markers for SVR, while the presence of the CC/CC/TT genotype combination indicates more precise responsiveness for CHC than the SNPs alone. The rs12979860-CC was associated with the lower secretion of CCL4 and CXCL10 suggesting that the levels of CCL4 and CXCL10 could be used as a hallmark for a good response to the Peg-IFN-α/RBV treatment. Our results confirm the literature and improve the knowledge about the effects of these *IFNL* polymorphisms in immune responses bringing insights towards the regulation of antiviral responses.

## Materials and Methods

### Patients and Clinical Specimens

We used samples from phase II/III randomized double-blind clinical trials. Blood from 740 treatment-naive CHC patients with viral genotypes (GT) 1, 2, and 3 (GT1, GT2/3) were collected and their baseline characteristics are shown in **Table 1**. The participants were from three regions of Brazil: South (88%), Southeast (10%), and Northeast (2%). Serum samples from 24 patients were collected before treatment begins, within the first hours of treatment, in the first, third, and 12^th^ weeks during treatment, and third week after the end of treatment. Also, serum samples from the clinical trial phase I and the 24 healthy volunteers were collected three times: 0 (before administration), 24, and 72 hours after Peg-IFN-α administration. All patients and volunteers in this study provided written informed consent. Both studies are registered in ClinicalTrials.gov under NCT01623336 and NCT01889849, respectively.

**Table 1:** Baseline demographics, clinical characteristics, viral genotype and outcome of CHC treatment with pegylated IFN-α and Ribavirin.

Characteristics	N	(%)	SVR	(%)	NR	(%)	P-value
All	707		441	(0.62)	266	(0.38)	
**Gender**							
F	376	(0.51)	247	(0.66)	129	(0.34)	0.063
M	331	(0.45)	194	(0.59)	137	(0.41)	
**Viral genotype**							
G1	344	(0.49)	171	(0.50)	173	(0.50)	<0.001
G2/3	363	(0.51)	270	(0.74)	93	(0.26)	
Ethnicity*							
Caucasoid	564	(0.80)	368	(0.65)	196	(0.35)	<0.001
Mestizo	91	(0.13)	55	(0.60)	36	(0.40)	
Black	52	(0.07)	18	(0.35)	34	(0.65)	
**Age range**							
19 – 42	186	(0.26)	117	(0.63)	69	(0.37)	0.982
43 – 57	366	(0.52)	228	(0.62)	138	(0.38)	
>58	155	(0.22)	96	(0.62)	59	(0.38)	
**Baseline liver fibrosis stage							
F0 – 1	359	(0.51)	240	(0.67)	119	(0.33)	0.022
F2	184	(0.26)	107	(0.58)	77	(0.42)	
F3 - 4	64	(0.09)	33	(0.52)	31	(0.48)	
**Baseline viral load (log10 IU/mL)**							
2,65 to 5,90	360	(0.51)	259	(0.72)	101	(0.28)	<0.001
>5,91	347	(0.49)	182	(0.52)	165	(0.48)	
**Treatment**							
Peg-IFNα2a	353	0,50	246	0,70	107	0,30	<0.001
Peg-IFNα2b	354	0,50	195	0,55	159	0,45	

*Self-declared ethnicity. **Some data is not available. Statistical analysis was performed by chi-square test, considering SVR and NR rates withing groups.

The study protocol conforms with the ethical guidelines of the 1975 Declaration of Helsinki and was approved by the Brazilian Ethics Committee for studies with human subjects (CONEP) with registration number CAAE 46065015.6.0000.5248. The patients were treated with combination therapy of 180 µg of Peg-IFNα2a (Pegasys^®^ from Roche Pharmaceutical) or Peg-IFNα2b (BIP-48 from Bio-Manguinhos/Fiocruz) (Costa et al., 2018) once per week and 1000 mg/day of RBV for patients up to 75 kg and 1,250 mg/day for those over 75 kg according to the Ministry of Health regulations (Ministério da Saúde, 2015). The so-called ‘2-log stopping rule’ was applied; i.e., discontinuation of therapy in patients with detectable HCV-RNA at the 12th week of treatment with a reduction of <2 log IU/mL in HCV-RNA. All patients were negative for hepatitis B and HIV. Liver fibrosis was scored according to the METAVIR scoring system as follows: F0 (no fibrosis), F1, F2 (fibrosis), F3, and F4 (cirrhosis) (Bedossa and Poynard, 1996). Patients were stratified into two groups based on sustained virological response (SVR) and non-responders (NR) treatment outcomes. The SVR was defined as an undetectable HCV RNA in serum more than 24 weeks after treatment termination; all other patients that were still viremic at this time and according to standard definitions were considered NR (Ministério da Saúde, 2015).

### Viral Load of the HCV

The viral load for all patients was determined before, during, and after the treatment. HCV quantification was performed by COBAS/Taqman HCV Test v2.0 (Roche). RNA was isolated with the High Pure System Viral Acid Kit (Roche), according to the manufacturer’s instruction. The real-time RT-PCR reaction set-up was carried out using an automated workstation (Janus, PerkinElmer). HCV detection occurred with the Nucleic Acid Testing (NAT) kit HIV/HCV/HBV from Bio-Manguinhos (ANVISA registration number 80142170025), performed according to the manufacturer’s instructions. Further details on the performance and interpretation of the results can be achieved in a previous study (Andrade et al., 2018).

### DNA and RNA Extraction

Whole blood was collected from each individual using RNA stabilization reagent (PAXgene^®^ blood tube) and anticoagulant EDTA tube. Genomic DNA extraction was performed from whole blood cells using the DNeasy Blood & Tissue extraction kit (QIAGEN, Germany). Genomic RNA was extracted using a commercial kit (PAXgene^®^ Blood RNA Kit, QIAGEN, Germany), according to the manufacturer’s instructions. Both RNA and DNA concentration and purity were checked in the spectrophotometer (Nanodrop ND 1000 Technologies), and RNA integrity was ascertained by 0.8% agarose gel electrophoresis. Samples were stored at -80 °C until use.

### Genotyping

All samples were genotyped for 12 SNPs encompassing *IFNL* locus using customized TaqMan SNP Genotyping Assays (Thermo Fisher, USA) and allele-specific probes labeled with a fluorescent dye (FAM and VIC). Three strategies to select the polymorphisms were followed: i) tag SNPs representative of the *IFNL3-IFNL4* region were selected by the analysis of minor allelic frequency (MAF>10%) and LD (r²>0.8) in the European, Native-American and African population from the 1000 Genomes Project (release 27, spanning 200Kb from the *IFNL3/4* genomic region) – rs11879005 and rs2099331 (located in the *IFNL2* gene); ii) the polymorphisms rs12979860, rs8099917, rs8105790, rs8109886, rs8113007, rs11881222, and rs12980275 were prioritized as candidate SNPs because they had been previously associated with Peg-IFN-α and ribavirin treatment outcome; and iii) three SNPs were selected due to its genomic position, possibly working as functional variants – the *missense* SNPs that leads to one aminoacid substitution – rs8103142 and rs4803221, and the 5’UTR (untranslated region) variant rs4803222. The specific location for each of the selected SNP and the allele frequencies is detailed in **Table S1**. Allelic discrimination and real-time PCR were performed in ABI StepOne Plus, following the manufacturer’s recommendations (Thermo Fisher, USA). Reactions used 20-40 ng of genomic DNA in a final volume of 20 µL, containing 10 µL of TaqMan Genotyping Master Mix (Thermo Fisher, USA) and 0.5 µL of each TaqMan probe.

### Multiplex Immunoassay

Twenty-seven cytokines: interleukins (IL) IL-1α, IL-1β, IL-2, IL-4, IL-6, IL-7, IL-9, IL-10, IL-13, IL-15, IL-17, IL-12(p70), interferon (IFN-γ), tumor necrosis factor (TNF), hematopoietic cytokine, GM-CSF, vascular endothelial growth factor (VEGF), Granulocyte-colony stimulating factor (G-CSF), fibroblast growth factor (b-FGF); chemokines, C-C motif chemokine ligands 2, 3, 4, 5 and 11 (CCL2/MCP-1, CCL3/MIP-1α, CCL4/MIP-1β, CCL5/RANTES, CCL11/Eotaxin) and C-X-C motif chemokine ligands 8 and 10 (CXCL8/IL-8 and CXCL10/IP-10) were measured using a Human Cytokine 27-plex assay kit (Bio-Rad, Hercules, CA, USA). Each assay was performed strictly according to the manufacturer’s protocol for serum or plasma samples, using recommended sample dilutions and standard curve concentrations, with all samples and standards assayed in duplicate. All measurements were performed on a MAGPIX^®^ System equipped with xPONENT v3.2, and data were analyzed using SoftMax Pro software version 5.4.

### Gene Expression of the *IFNL3/4* by Real-Time RT-PCR

In brief, cDNA conversion reactions were conducted with oligo (dT) primers and SuperScript II™ reverse transcriptase (Thermo Fisher, USA), following the manufacturer’s instructions. Next, RT-qPCR was used to measure interferon lambda transcripts using TaqMan^®^ Gene Expression Master Mix (Thermo Fisher, USA). A primer/probe predesigned by the IDT (Integrated DNA Technologies) of *IFNL1* (NM_172140 - Hs.PT.56a.38564463.g) and *IFNL2* (NM_172138-Hs.PT.39a.22214856.g) was used. Primer/probe sequences for specific detection of *IFNL3* and *IFNL4* were custom-designed based on the NCBI human genome assembly (NCBI36/hg18). Reference genes beta-2-microglobulin (B2M) and 18S were quantified using predesigned IDT probe-assays. The expression of 30 target genes and 3 normalizing genes (**Table S2**) was performed by medium-throughput quantitative qPCR using the microfluidic system Biomark (Fluidigm, CA), the primers used are detailed in **Table S2**. The analysis was performed from the real-time fluorescence accumulation data of each sample (ΔRn), using the logistic function adjustment of four parameters to represent each amplification curve by the library of qPCR (R Development Core Team, 2009) version 2.922.

### Statistical Analysis

In the genetic association study, all the analyses were performed in R environment version 3.4.0 (http://www.r-project.org) using the packages “genetics” v. 1.3.8.1, “SNPassoc” v. 1.9-2”, and “epiDisplay” v. 3.2.2” [R Core Team, (2018)]. First, we compared the differences in means or medians for the clinical and demographic variables concerning the treatment outcome to see the variables that could influence the association analysis. Then, to test for the association of the SVR with the *IFNL3/4* SNPs, we used the Odds ratio (OR), which were estimated together with the 95% confidence intervals (CI) by logistic regression, using Bonferroni procedure to adjust for multiple comparisons. An OR>1 was associated with non-response (NR), and OR<1 was associated with a lower chance to NR. Analyses were adjusted for non-genetic variables (viral genotype, ethnicity, fibrosis grade, baseline viral load, and drug) that were significantly different among the groups of patients by the chi-square test (p-value<0.05). Haploview v. 4.2 was used to estimate pairwise linkage disequilibrium (LD) between SNPs and haplotype construction for all data in this study, considering *r^2^*>0.8 as strong LD (Barrett et al., 2005). For the functional analysis of cytokine detection in serum, linear values were transformed to log (base 10) and statistical significance was considered if two-sided *P*-values were less than 0.05. The statistical analyses of the multiplex assay were performed in GraphPad 5, applying one-way ANOVA, Kruskal-Wallis test, and Dunn’s Multiple Comparison Test to compare the levels of cytokine between the different treatment times. Two-way ANOVA followed by the Sidak post-test was used to compare the levels of cytokines between the genotypes across different time periods. Finally, gene expression analyses were also performed in the R 3.4.0 environment. For relative gene expression quantification, the fluorescence accumulation data of each sample were used to fit four-parameter sigmoid curves using the R package “qPCR” (Ritz and Spiess, 2008). For each amplification, the cycle of quantification was determined as the maximum of the second derivative of the fitted sigmoid curve, and the efficiency as the ratio between the fluorescence of the cycle of quantification and the fluorescence of the cycle immediately preceding that. For each gene, the efficiency was estimated by the mean of all efficiencies for each amplification reaction of that gene. Reference genes used in normalization between the different amplified samples were selected by the geNorm method (Vandesompele et al., 2002). Normalization factors were estimated for each sample using the geometric averaging of the most stable selected reference genes (Vandesompele et al., 2002). After normalization, the statistical analysis of gene expression for comparisons between times and genotypes were the same as described for the multiplex assays.

## Results

### Baseline Features Associated With the SVR

The epidemiological characteristics and treatment outcomes of the CHC patients, viral genotype, and combined therapy (Peg-IFN-α/RBV) are shown in **Table 1**. Of the 740 patients included in the study, 33 (4.5%) did not follow the trial protocol or failed to complete the treatment and were excluded from the study. Among the 707 subjects, 62% had obtained an SVR (characterized as the undetectable HCV RNA levels among 24-weeks after the treatment conclusion), while 38% were considered non-responders (NR). The demographic and clinical characteristics of the patients were presented in **Table 1**. Comparing patients responders and non-responders, the age range (p=0.982) and gender (p=0.063) showed no difference between patients who achieved SVR and NR, as shown in **Table 1**. However, patients infected with GT1 presented a lower percentage of SVR (50%) when compared to 78% of patients with GT2/3 (p<0.001). Black patients had a lower rate of SVR (35%) than Caucasoid (65%) and Mestizo (60%) (p<0.001). Individuals with fibrosis stage F3 and F4 had lower capacity to reach SVR compared to patients with stage F0, F1 and F2 (p=0.022). Before the start of the treatment, patients with viral load <5.90 log10 IU/mL obtained higher SVR rates (72%), while patients above 5.91 log10 IU/mL presented 52% (p<0.001). Between the two biopharmaceuticals, a significant difference was seen with p<0.001, thus the patients treated with Peg-IFNα2a reached an SVR rate of 70% compared to 55% treated with Peg-IFNα2b, without taking into account the viral genotype (**Table 1**).

### *IFNL3/4* SNPs Are Associated With the Treatment Outcome of CHC

**Figure 1:**
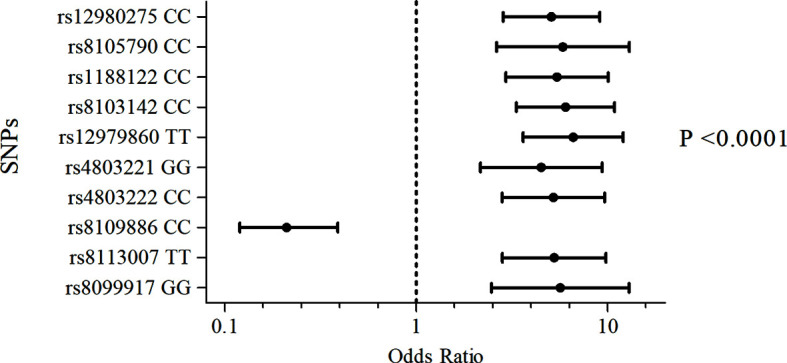
Association of the SNPs of the *IFNL3/IFNL4* region with the outcome of Peg-IFN-α/RBV treatment. Logistic regression models included viral genotype, ethnicity, fibrosis, baseline viral load, and type of treatment as categorical covariates. P-values were adjusted by Bonferroni.

The frequency of the genotypes and minor allele carriers of the 12 SNPs were evaluated in 707 subjects to test for association with the treatment outcome (SVR or NR) (**Table S2**). The strongest signal for the non-response association was generated by the TT genotype of rs12979860, which showed an *Odds Ratio* of 6.6 after adjusting for confounding factors (viral genotype, ethnicity, fibrosis, baseline viral load, and type of treatment), as shown in **Table S3** and **Figure 1**. In contrast, minor allele carriers to SNP rs8109886 showed a significant OR, lower than 1, (**Figure 1**), indicating that patients carrying the allele C had higher chances of sustained virological response after the treatment. All other SNPs presenting the allele exhibiting lower frequency exhibited OR higher than 1, suggesting a higher chance for NR (**Table S3**). The covariates adjustments did not impact OR and p-values that remained significant for ten tested SNPs (**Figure 1**).

### rs12979860, rs8109886, and rs8099917 Are Tag SNPs of the *IFNL3/4* Gene

We also analyzed the linkage disequilibrium (LD) between the associated SNPs among all the patients. We found a strong LD between rs12979860, rs12980275, rs11881222, rs8103142, rs8113007 and rs4803222 that formed one major bin with r^2^>0.75. Another bin of SNPs in strong LD (r^2^ = 0.85) was composed by rs8105790, rs4803221, and rs8099917. A weak-to-moderate LD was observed for the SNP rs8109886 with all others. There was no LD for SNPs rs11879005 and rs2099331, as shown in **Figure S1**. No marked LD differences between the SVR and NR groups were observed (data not shown). It was shown that the LD value of these SNPs was similar to European and Amerindian populations retrieved from the 1000 Genome Project database (data not shown). Thus, we could use rs12979860, rs8109886, and rs8099917 as tag SNPs of the *IFNL3/4* locus in this cohort.

### Genotype Combination CC/CC/TT (rs12979860/rs8109886/rs8099917) as the SVR Outcome Predictor

**Figure 2:**
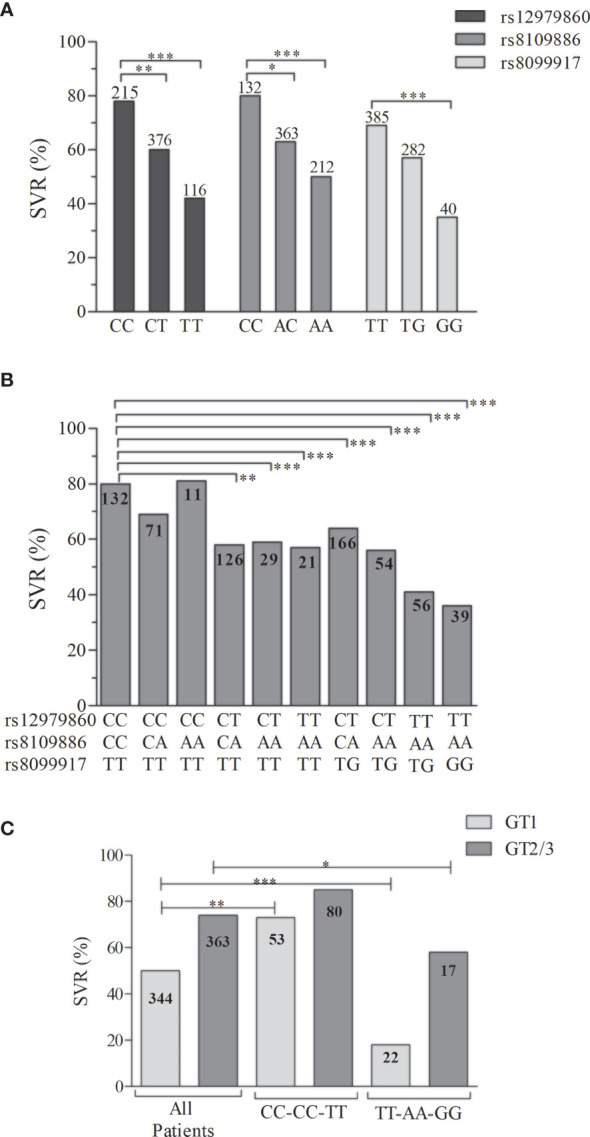
Distribution of the SVR rates from the patients with CHC treated with Peg-IFNα. The distribution is stratified by **(A)** the genotypes of the SNPs rs12979860, rs8109886, and rs8099917, **(B)** the genotypic combination of those SNPs, and **(C)** the genotypic combination of the opposite prognostic markers. The column numbers represent the available individuals in each group. The SVR percentage were compared among groups by the Fisher’s exact test (*P < 0.05, **P < 0.001, ***P < 0.0001).

The genotype frequencies of the SNPs rs12979860, rs8109886, and rs8099917 within the treated patients were evaluated according to the SVR outcome. **Figure 2A** showed that the predictor genotypes for those SNPs exhibited the following rates: rs12979860-CC, 78%; rs8109886-CC, 80%; and rs8099917-TT, 69%. However, since the total number of responsive individuals per genotype varies, a better description of the responsive phenotype can be achieved by combining the genotypes (**Figure 2B**). The arrangement of the SNPs rs12979860, rs8109886, and rs8099917, which tag different *IFNL3/4* bins provide a genotype combination that could capture the information from the entire genomic region. Besides, data retrieved from the 1000 Genomes (“Homo sapiens - Ensembl genome browser 99”, n.d.) indicate that the arrangement of rs12979860-C, rs8109886-C, and rs8099917-T is the most frequent haplotype in Europeans, while rs12979860-T, rs8109886-A, and rs8099917-T is the most frequent haplotype in Africans, confirming that all the three SNPs define an extended block that also indicates a dependency between these markers. Thus, evaluating the probability of the genotype combination to provide a more precise prediction of the treatment outcome, our findings suggest that combining the SNPs rs12979860, rs8109886, and rs8099917 increases the precision of the SVR prediction (**Figure 2B**). Furthermore, SVR depends on the presence of the C/C/T haplotype. Thus, when 2 haplotypes are presented (CC**/**CC**/**TT genotypes), 80% of responsiveness is detected. However, the combination of CC**/**CA**/**TT drops down the SVR to 69%. Finally, in the absence of the CC**/**CC**/**TT genotype combination, which is seen among the TT**/**AA**/**TG and TT**/**AA**/**GG combinations, a poor prognosis of responsiveness (36-41%) is observed (**Figure 2B**). In addition, when stratified by viral genotype an increase among responders for carriers of CC/CC/TT genotype was also observed mainly to GT1 (**Figure 2C**). Together, our data suggest that we might improve the precision of responsiveness within patients by including 3 SNPs of the *IFNL3/4* region to the genotype combination.

### Expression of Type I and III IFN Signaling Pathways Genes

**Figure 3:**
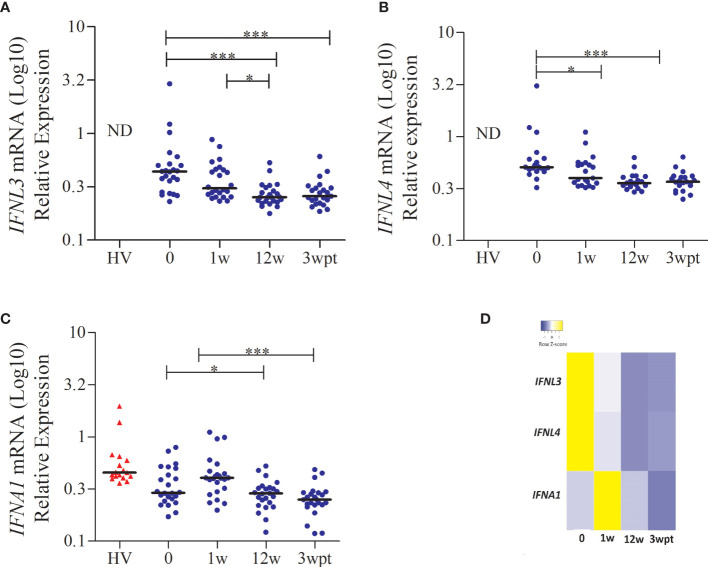
*IFNL3, IFNL4, and IFNA1* gene expression in patients with CHC during the treatment with Peg-IFNα and RBV. **(A)** *IFNL3*; **(B)** *IFNL4*; **(C)** *IFNA1* and **(D)** Heat map showing the antagonistic effect between the expression of *IFNL3/4* and *IFNA1*. Statistical analysis was performed by the Kruskal-Wallis test and Dunn’s multiple comparison test (*P < 0.01, **P < 0.001, ***P < 0.0001). ND, not detected; HV, healthy volunteer; w, week; wpt, week post-treatment.

In a subgroup analysis, we included individuals who were part of phase I (healthy subjects) and phase II/III (patients) clinical trials for Peg-IFNα + RBV. We enrolled 24 patients, and whole blood gene expression of the type I and III IFNs and ISGs was analyzed. Before treatment, the expression of *IFNA1* and *IFNAR1* presented low levels and increases one week after treatment, in contrast with *IFNL3, IFNL4*, and the ISGs (*RIGI*, *TICAM*, *IRF3*, *IRF7*, *IRF9*, *IFI6*, *IFI16*, *IFI44*, *IFI35*, *IFIH1*, *IFIT1*, *IFIT2*, *IFIT5*, *IFITM1*, *IFITM3*, *ISG15*, *OAS1*, *OAS2*, *OAS3*, *OASL*) (**Figure S2**). To better understand the dynamics between type I and III IFNs expression in chronic hepatitis C patients, we also quantified the expression of *IFNA1, IFNL1, IFNL2, IFNL3* and *IFNL4* in 24 healthy volunteers. In this group, the four genes of IFN-λ were below the detection limit. As shown in **Figures 3A**, **B**, higher levels of *IFNL3* and *IFNL4* mRNA were seen in patients before treatment. Applying statistical analysis comparing the different times, higher levels of *IFNL3* and *IFNL4* mRNA were seen in patients before treatment, followed by a decrease in the 12^th^ week in both SVR and NR patients. On the other hand, *IFNA1* expression increases in the first week of treatment (**Figure 3C**), suggesting an antagonistic effect between the expression of *IFNL3/4* and *IFNA1*, as represented in **Figure 3D**.

### Expression of Type I and III IFN Signaling Pathways Genes by rs12979860 Genotypes

**Figure 4:**
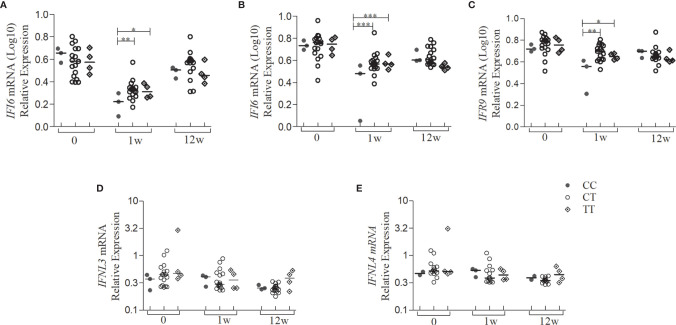
Gene expression levels of the type I IFN signaling pathway and type III IFN of patients with CHC treated with Peg-IFNα/RBV stratified by the SNP genotype rs12979860. Quantification of the expression of the IFN type I signaling pathway genes from whole blood of patients with CHC (n = 24) treated with Peg-IFNα/RBV at times 0, 1, and 12 weeks of treatment. **(A)** *IFI6*; **(B)** *IFI16*; **(C)** *IRF9;* **(D)** *IFNL3*; **(E)** *IFNL4* mRNA expression. Expression of other genes analyzed did not present statistically significant results. Statistical analysis was performed by Kruskal-Wallis test and Dunn’s multiple comparison test by each genotype in the same group and between the groups 0, 1w, and 12w (*P < 0.01, **P < 0.001, ***P < 0.0001). w, week; wpt, week post-treatment.

Since we had a small sample size in the subgroup analysis, we selected only the SNP rs12979860 for the genotype-phenotype association. Both *IFNA1*, *IFNL3*, and *IFNL4* showed no significant differences between genotypes (CC-CT-TT) as they did show between the weeks of treatment (**Figure 3**). It was observed that in the first week of treatment, the homozygote CC patients for rs12979860 had lower *IFI6*, *IFI16*, and *IRF9* gene expression levels, compared to patients’ carriers of risk allele T, as shown **Figures 4A–C** (p<0.05). However, stratifying the patients accordingly to the genotype, no differences for *IFNL3* or *IFNL4* expressions were observed (**Figures 4D, E**).

### Effects of rs12979860 on CCL4 and CXCL10 Levels

**Figure 5:**
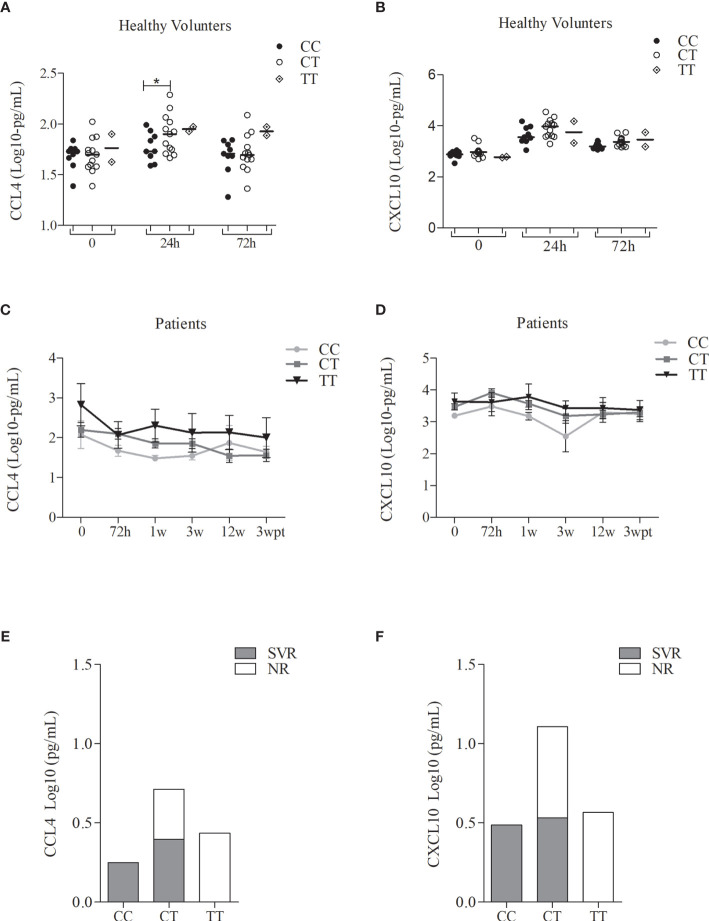
Serum levels of the CCL4 and CXCL10 of the patients with CHC treated with Peg-IFNα/RBV and healthy volunteers who received one dose of Peg-IFNα stratified by rs12979860 genotypes. **(A)** CCL4 in healthy volunteers; **(B)** CCL4 in patients; **(C)** CCL4 levels before treatment in patient responders and non-responders; **(D)** CXCL10 in healthy volunteers; **(E)** CXCL10 in patients; **(F)** CXCL10 levels of the first week of treatment in patient responders and non-responders. Number of the genotypes in patients CC (n = 3), CT (n = 17) and TT (n = 4) and in health volunteers CC (n = 9), CT (n = 13) and TT (n = 2). Data were compared by the two-way ANOVA, plus post-hoc comparisons adjusted by the Sidak procedure (*P < 0.05). Results are represented as medians. h, hours; w, week; wpt, week post-treatment. Serum levels of other cytokines analyzed did not present statistically significant results.

In parallel, 27 cytokines were evaluated in serum samples of the same subgroup of patients and healthy volunteers, aiming to measure the effect of the treatment on the levels of these cytokines and to detect a possible genotype-phenotype correlation. The group of patients with CHC presented high levels of serum cytokine before the treatment while a decrease were observed throughout the treatment for the majority of the cytokines (**Figure S3**). Regarding IFN-α serum levels, it was observed increased levels along treatment, followed by a decrease after treatment (**Figure S4**). In the group of healthy volunteers, a significant difference was observed for the CCL4 and CXCL10 chemokine levels, after 24h of Peg-IFNα administration, characterizing the biopharmaceutical induction (data not shown). The levels of these chemokines also showed significant differences along with the treatment. CCL4 significantly decreases in the first week of treatment until the end of it, as shown in **Figure S5A**. Also, an increase of CXCL10 was observed right after the beginning, with a peak in 72h after treatment, and decreased throughout the treatment (**Figure S5B**). However, a group of patients, who also presented higher CCL4 production, preserved high levels of CXCL10 until the end of the treatment (**Figures S5A, B**). When we stratified the CCL4 levels in healthy volunteers by the genotypes of the SNP rs12979860, we observed significant differences between CC and CT genotypes, but not for CXCL10 production (**Figures 5A–D**). Among patients (SVR or NR), we observed that individuals with CC genotype presented lower levels of CCL4 and CXCL10 in comparison with the TT genotype, independent of the time of treatment (**Figures 5B–E**). Higher levels of CCL4 and CXCL10 were observed in NR patients in comparison with SVR patients (**Figures 5C–F**). Therefore, the negative modulation of these chemokines can be correlated with favorable genotype therapy outcome, since the highest CCL4 and CXCL10 levels were found in patients with the rs12979860-T allele, which is associated with the NR outcome.

## Discussion

The SNPs in the region of IFN-λ are associated with the standard treatment response for chronic hepatitis C as well as with the spontaneous HCV clearance (Thomas et al., 2009), as *IFNL3* SNPs, are considered the strongest predictors upon antiviral therapy response (Cavalcante and Lyra, 2015). Here, we provide a more precise prediction of SVR in the treatment of CHC with Peg-IFN-α/Ribavirin using a genotype combination of rs12979860, rs8109886, and rs8099917 SNPs among Brazilians. Interestingly, none of these SNPs are located in the coding region of IFNλ3, and the mechanism by which these variants affect the response to CHC therapies is not completely understood. The rs12979860 SNP, for example, is localized in the intronic region of the *IFNL4* gene and displaying a high LD (r^2^) with the exonic indel rs368234815- ΔG >TT of *IFNL4*. The (TT) dinucleotide insertion disrupts the *IFNL4* open reading frame and tags along with the rs12979860 C allele. The extended haplotype rs368234815-TT/rs12979860-C is associated with increased HCV clearance (Prokunina-Olsson et al., 2013), and suggestive signatures of positive selection and pseudogenization (Key et al., 2014).

In this study, we also evaluated SNPs in the *IFNL3/4* region among Brazilians, an admixed population of three paternal lineages consisting of Native-American, European and African ancestries. Other studies with CHC patients treated with Peg-IFN-α/Ribavirin from different regions in Brazil focused on the SNPs rs12979860 and rs8099917, evidencing that CC and TT genotypes, respectively, presented favorable treatment outcomes (Ge et al., 2009; Thomas et al., 2009; Cavalcante et al., 2012; Ramos et al., 2012; Moreira et al., 2016; Rizzo et al., 2016). Here, we confirmed the association of several SNPs with the responsiveness for treatment, highlighting the rs12979860 as the strongest predictor in obtaining SVR in patients treated with Peg-IFNα. The rs8109886-CC SNP exhibited the highest SVR rate among patients, although the rs8109886-CC patients also carried the predictive genotypes rs12979860-CC and rs8099917-TT, clarifying the phenotypic effect of this SNP. Thus, we suggest that rs8109886 is an important tag predictive marker in the Brazilian population and should be used together with the SNPs rs12979860 and rs8099917 before customizing the treatment based on Peg-IFNα/RBV therapy for patients infected with HCV, who would need interferon-based therapy.

**Figure 6:**
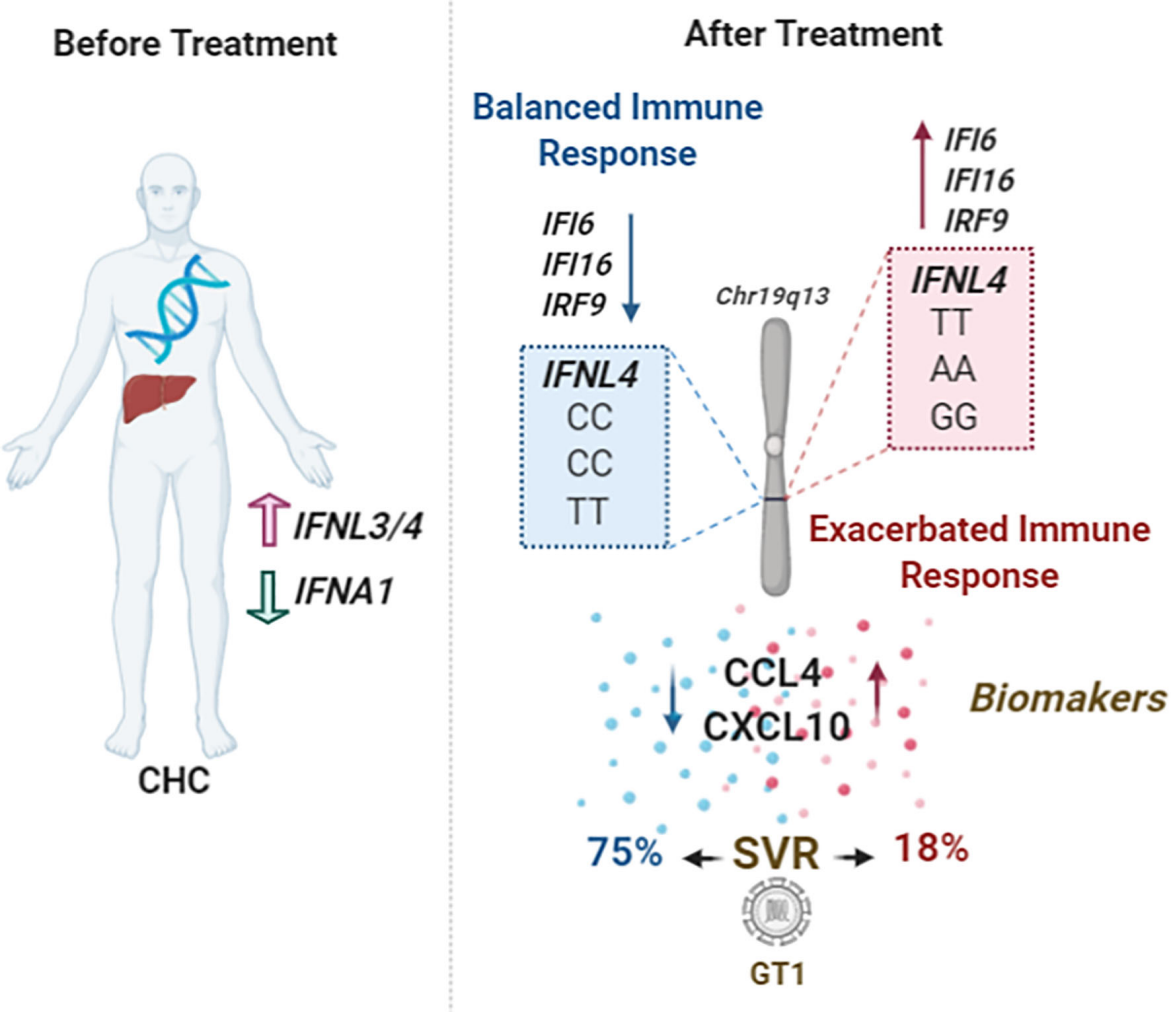
Schematic model showing the main findings of this study. Patients with CHC present high levels of cytokines before treatment and display an antagonistic effect between *IFNL3/4* and *IFNA1* expression levels. After treatment, patients with the genotypic combination of SNPs rs12979869-CC/rs8109886-CC/rs8099917-TT can induce a balanced immune response, presenting lower levels of ISGs, IFI6, IFI16, and IRF9, and low serum levels of the chemokines CCL4 and CXCL10. These patients can increase the SVR prediction, especially those infected with GT1, contrary to the patients with genotypes rs12979869-TT/rs8109886-AA/rs8099917-GG.

CHC patients are classified with a positive or negative prognosis based on several factors such as age, fibrosis index, and ethnicity. We revealed that the genotype combinations may improve SVR prediction and may elevate the percentage of SVR to 75% for HCV GT1, and patients with the risk genotype (rs12979860TT, rs8109886-AA rs8099917-GG) only 18% obtained SVR, compared to no combination of predictive parameters (**Figure 6**). It was also possible to observe that the genotype parameters exceeded the expectation of a good treatment outcome, regardless of the risk classification. Therefore, the genotypic combination might be a strategic approach in the custom of Peg-IFNα/RBV therapy for some patients.

Moreover, it is likely that the combination of rs12979860-CC rs8109886-CC, and rs8099917-TT genotypes could provide a better prediction of favorable innate responses in other viral diseases since IFN-lambda balances type-I IFNs and chemokines, **Figure 6**. Our data suggest differential expression of *IFI6*, *IFI16*, and *IRF9* genes when stratified by rs12979860. This corroborates with the data showing that high expression of ISGs was associated with a lower rate of SVR (Pfeffer et al., 2014). Despite the analyzed samples here coming from whole blood, with a poor IFN-λ expression, the antagonism between IFN-λ and type-I IFNs was observed and have been already indicated before providing an interesting mechanistic way in how *IFNL* region SNPs regulate innate immunity during viral infections (Bordi et al., 2015; Azamor et al., 2019). We can suggest that the role of genetically regulated levels of IFN-λ could be fine-tuning the levels of IFN-α and necessary ISGs, where optimal levels can control viral replication and inflammatory progression while high levels would not be as efficient.

Chemokines and their receptors are key players in HCV-associated liver inflammation (Thompson et al., 2010; Hayes et al., 2011; Cavalcante et al., 2012) and are involved with the immunopathogenesis of chronic hepatitis (Lin et al., 2017). Furthermore, patients responding to the treatment with Peg-IFN-α2b presented lower CXCL10 levels than the non-responder group (Gong et al., 2015). Additionally, most of the studies reported an increase of CCL3 and CCL4 levels in serum and liver (Zeremski et al., 2007), but lower pre-treatment levels of CCL4 can reliably predict a favorable treatment outcome (Zhang et al., 2015). Thus, levels of CCL4 and CXCL10 may predict an effective HCV clearance, underlining that the balance of those chemokines levels is associated with a good prognosis (Florholmen et al., 2011; Kurelac et al., 2012; Lin et al., 2017). Our data demonstrated a similar profile in which the genotype rs12979860 differentially induced CCL4 and CXCL10, confirming the results from the literature. High CXCL10 levels were associated with treatment unresponsiveness and were higher among rs12979860 T carriers. On the other hand, CCL4 levels presented a higher peak and then decreased after starting the treatment. Besides, individuals with rs12979860-CC genotype maintained the low-level of CCL4 throughout the treatment, which indicates that these chemokines are a good readout of the genetic markers and could also be used to follow up on treatment outcomes. Indeed, this result corroborates the findings that a higher baseline serum level of these chemokines was independently associated with non-response.

In summary, our data showed the regulation of the ISGs and pro-inflammatory chemokines production by the rs12979860-CC genotype. In addition, we suggested that the combination of the genotypes rs12979860, rs8109886, and rs80999917 are more precise predictors of the treatment outcome in admixed Brazilians. The combination CC/CC/TT (rs12979860/rs8109886/rs8099917) genetically regulates the innate and inflammatory responses by modulating the secretion of type III IFNs and fine-tuning type I IFNs levels.

## Data Availability Statement

The raw data supporting the conclusions of this article will be made available by the authors, without undue reservation.

## Ethics Statement

The studies involving human participants were reviewed and approved by Brazilian Ethics Committee for studies with human subjects (CONEP) with registration number CAAE 46065015.6.0000.5248. The patients/participants provided their written informed consent to participate in this study.

## Author Contributions

AMVS, LEAA, PCCN, MOM, and DCM conceived the study design. DCM, MLSM, and MOM funded the study. AMVS, ACMA, JRX, EMS, MLSM, RPM, HNC, GBS, and PDP participated in data curation. AMVS, TA, LEAA, CB, JS, AFS, MM, and EFA participated in the methodology. AMVS, LEAA, FSGK, MRA, and TLC performed the statistical analysis. LEAA, LRBS, TLC, TA, DCM, and MOM supervised and validated the analysis. AMVS wrote the original manuscript. AMVS, LEAA, LRBS, OCLB, DCM, and MOM provided critical reviewing and helped shape the final manuscript. All authors contributed to the article and approved the submitted version.

## Funding

This work was supported by Instituto de Tecnologia em Imunobiológicos Bio-Manguinhos and Fundação Oswaldo Cruz, Conselho Nacional de Desenvolvimento Científico e Tecnológico (CNPq), Coordenação de Aperfeiçoamento de Pessoal de Nível Superior (CAPES) and Fundação de Amparo à Pesquisa do Estado do Rio de Janeiro (FAPERJ).

## Conflict of Interest

The authors declare that the research was conducted in the absence of any commercial or financial relationships that could be construed as a potential conflict of interest.
