# Supplementary material for: Interferon-lambda 3 and 4 Polymorphisms Increase Sustained Virological Responses and Regulate Innate Immunity in Antiviral Therapy With Pegylated Interferon-Alpha
Source: Front Cell Infect Microbiol. 2021 Jul 7;11:656393. doi: 10.3389/fcimb.2021.656393 (PMC8294038; doi:10.3389/fcimb.2021.656393)
Supplement: Supplementary file 1 [file DataSheet_1.docx]

***Supplementary Material***

**Table S1**. Candidate SNPs of the *IFNL3/4* region and allelic frequency in the European, Native-American, and African populations from the 1000 Genomes Project.

| **Genomic region** | **Location (chr19.3)** | **Gene** | **SNP** | **A>a** | **Allele Frequency (%)** | | |
| --- | --- | --- | --- | --- | --- | --- | --- |
|  |  |  |  |  | **European** | **Native-American** | **African** |
| Upstream | 39220263 | *IFNL3* | rs11879005 | C>T | 59/41 | 64/36 | 80/20 |
| Upstream | 39241143 | *IFNL3* | rs12980275 | A>G | 70/30 | 60/40 | 44/56 |
| Upstream | 39241861 | *IFNL3* | rs8105790 | T>C | 83/17 | 70/30 | 82/18 |
| Intron | 39244283 | *IFNL3* | rs11881222 | T>C | 71/29 | 63/37 | 69/31 |
| Missense | 39244466 | *IFNL3* | rs8103142 | T>C | 69/31 | 60/40 | 30/70 |
| Intron | 39248147 | *IFNL4* | rs12979860 | C>T | 69/31 | 60/40 | 33/67 |
| Missense | 39248489 | *IFNL4* | rs4803221 | C>G | 83/17 | 70/30 | 84/16 |
| 5’UTR | 39248713 | *IFNL4* | rs4803222 | G>C | 70/30 | 63/37 | 75/25 |
| Downstream | 39252122 | *IFNL4* | rs8109886 | C>A | 57/43 | 50/50 | 16/84 |
| Downstream | 39252463 | *IFNL4* | rs8113007 | A>T | 71/29 | 64/36 | 73/27 |
| Downstream | 39252525 | *IFNL4* | rs8099917 | T>G | 83/17 | 72/28 | 96/4 |
| Downstream | 39273910 | *IFNL2* | rs2099331 | T>G | 76/24 | 74/26 | 51/49 |

Location is based on the GRCh38 assembly. Abbreviations: A=allele 1; a=allele 2. Allele frequency was showed by percentage and displayed as A/a.

# **Table S2**. The list of primers used in this study.

| **Genes** | **Sense (5' → 3')** | **reverse (5' → 3')** |
| --- | --- | --- |
| *FOXP3* | AGAAGCAGCGGACACTCAAT | CTTGTGCAGACTCAGGTTGTG |
| *IFI16* | TTGAATTGGCACCGAAAAGT | TTTCTTGTTTTTCCTGGTCTTGA |
| *IFI35* | GGTTTGCTAGGGATGGAGTG | ACTGGCTGCGACCTGATCT |
| *IFI44* | GGGAGCTGGACCCTGTAAA | TTTCCTCCCTTAGATTCCCTATT |
| *IFI6* | CTGGTCTGCGATCCTGAATG | ATACTTGTGGGTGGCGTAGC |
| *IFIH1* | CTAGCCTGTTCTGGGGAAGA | CACTTCTTTTGCAGTGCTTTGT |
| *IFIT1* | AGAACGGCTGCCTAATTTACAG | TCAGGCATTTCATCGTCATC |
| *IFIT2* | GCACTGCAACCATGAGTGA | GGAGTTTTCTCCCTCCATCAA |
| *IFIT5* | CATGAGTGAAATTCGTAAGGACAC | ACCTCAAACAGATCAATGTCTTC |
| *IFITM1* | TTCTTGAACTGGTGCTGTCTG | CCCAGATGTTCAGGCACTTG |
| *IFITM3* | CGCCTACTCCGTGAAGTCTA | GGGATGACGATGAGCAGAAT |
| *IFNA1* | GCCATCTCTGTCCTCCATGA | TGGTAGAGTTCGGTGCAGAAT |
| *IFNAR* | TGCTGCGAAAGTCTTCTTGA | CGATTTGTTCCTCAGAAGTTGA |
| *IRF3* | TCGAGGTGACAGCCTTCTAC | GCCTCACGTAGCTCATCACT |
| *IRF7* | TGATGCTGCGGGATAACTC | CCTGTGGTGGTGGGACAG |
| *IRF9* | TCCAGCCATACTCCACAGAA | AGGCTCTACACCAGGGACAG |
| *ISG15* | GAGAGGCAGCGAACTCATCT | CCAGCATCTTCACCGTCAG |
| *OAS1* | GGCTGAATTACCCATGCTTTA | ATCGTCGGTCTCATCGTCTG |
| *OAS2* | GTTGGTGTTGGCATCTTCTG | CCACAACTTCCTGGTGTCTG |
| *OAS3* | TGTTGGAGACTTCCTGAAACAG | ATGCAGCACAGGGCAGAT |
| *OASL* | AAATTTCTGCCCATCCTTCAG | TGGCTTTCACATACTGCTGGTA |
| *PDL1* | AGGGAGAATGATGGATGTGAA | CGTCTCCTCCAAATGTGTATCA |
| *RIGI* | AAGCCTTGGCATGTTACACAG | GGCTTGGGATGTGGTCTACT |
| *RNASEL* | ACCTGGGCCTTCTGAACATT | CGGATGAACTTTAGCAGATCAC |
| *SOCS3* | AAGGACGGAGACTTCGATTC | GGAAACTTGCTGTGGGTGAC |
| *STAT2* | CCAGATTTGCCCTGTGATCT | AAACCTCATCCACGGTGTTC |
| *STING* | ATATCTGCGGCTGATCCTGC | CATCTGCAGGTTCCTGGTAGG |
| *TBK1* | GAAGGGCGACGCTTAGTCTT | ACCCCTGTTATTGCCTTAGCC |
| *TBX21* | CCAACAATGTGACCCAGATG | TCTCCGTCGTTCACCTCAA |
| *TICAM1* | GGATCCCTGATCTGCTTGG | GAGGTGGTGAAGGCATGTTC |
| *18S* | AGGATGAGGTGGAACGTGTG | CTTCACGGAGCTTGTTGTCC |
| *B2M* | AGATAGTTAAGTGGGATCGAGACA | ATTAAAAAGCAAGCAAGCAGAA |
| *GAPDH* | AGTGATGGCATGGACTGTGGTCAT | CAACAGCCTCAAGATCATCAGCAA |

**Table S3.** Frequency of the genotypes and minor allele carries of each *IFNL3/4* SNPs and association with the treatment outcome.

| **SNPs** | **Genotypes Carriers** | **Total Frequency** | | **Unadjusted Model** | | **Adjusted Model** | |
| --- | --- | --- | --- | --- | --- | --- | --- |
|  |  | **441** | **266** |  |  |  |  |
|  |  | **SVR = n (%)** | **NR= n (%)** | **OR** | **P-value** | **OR** | **P-value** |
|  | T/T | 171 (74.0) | 60 (26.0) | Baseline | | Baseline | |
|  | T/C | 219 (60.5) | 143 (39.5) | 1.9 (1.3-2.7) | 4,62E-07 | 2.6 (1.7-4.0) | 2,38E+06 |
| **rs12980275** | C/C | 51 (44.7) | 63 (55.3) | 3.5 (2.2-5.6) |  | 5.1 (2.9-9.1) |  |
|  | T/T | 171 (74.0) | 60 (26.0) | Baseline | | Baseline | |
|  | Carrier C | 270 (56.0) | 206 (43.3) | 2.2 (1.5-3.1) | 6,06E-06 | 3.0 (2.0-4.6) | 1,10E+07 |
|  | T/T | 255 (69.3) | 113 (30.7) | Baseline | | Baseline | |
|  | T/C | 171 (58.4) | 122 (41.6) | 1.6 (1.2-2.2) | 1,86E-06 | 2.2 (1.49-3.3) | 4,06E-07 |
| **rs8105790** | C/C | 15 (32.6) | 31 (67.4) | 4.7 (2.4-9.0) |  | 5.8 (2.6-13.0) |  |
|  | T/T | 255 (69.3) | 113 (30.7) | Baseline | | Baseline | |
|  | Carrier C | 186 (54.9) | 153 (45.1) | 1.9 (1.3-2.5) | 7,47E-05 | 2.5 (1.7-3.7) | 1,29E-06 |
|  | T/T | 185 (72.3) | 71 (27.7) | Baseline | | Baseline | |
|  | T/C | 218 (60.6) | 142 (39.4) | 1.7 (1.2-2.4) | 1,08E-06 | 2,4 (1.6-3.7) | 3,37E-08 |
| **rs11881222** | C/C | 38 (41.8) | 53 (58.2) | 3.6 (2.21-6.0) |  | 5,4 (2.9-10.1) |  |
|  | T/T | 185 (72.3) | 71 (27.7) | Baseline | | Baseline | |
|  | Carrier C | 256 (56.8) | 195 (43.2) | 2.0 (1.43-2.8) | 3,55E-05 | 2.8 (1.9-4.3) | 3,20E-07 |
|  | T/T | 157 (76.2) | 49 (23.8) | Baseline | | Baseline | |
|  | T/C | 229 (60.9) | 147 (39.1) | 2.1 (1.4-3.0) | 1,85E-08 | 2.87 (1.8-4.5) | 1,50E-09 |
| **rs8103142** | C/C | 55 (44.0) | 70 (56.0) | 4.1 (2.5-6.6) |  | 6.0 (3.3-10.9) |  |
|  | T/T | 157 (76.2) | 49 (23.8) | Baseline | | Baseline | |
|  | Carrier C | 284 (56,7) | 217 (43.3) | 2.5 (1.7-3.5) | 6,47E-07 | 3.4 (2.2-5.3) | 1,67E-08 |
|  | C/C | 165 (76,7) | 50 (23.3) | Baseline | | Baseline | |
|  | C/T | 227 (60.4) | 149 (39.6) | 2.2 (1.5-3.2) | 1,89E-09 | 3.0 (1.9-4.7) | 2,59E-10 |
| **rs12979860** | T/T | 49 (42.2) | 67 (57.8) | 4.5 (2.8-7.3) |  | 6.6 (3.6-12.1) |  |
|  | C/C | 165 (76.7) | 50 (23.3) | Baseline | | Baseline | |
|  | Carrier T | 276 (56.1) | 216 (43.9) | 2.6 (1.8-3.7) | 9,82E-08 | 3.5 (2.3-5.5) | 4,32E-09 |
|  | C/C | 244 (69.3) | 108 (30.7) | Baseline | | Baseline | |
|  | C/G | 178 (58.7) | 125 (41.3) | 1.6 (1.2-2.2) | 8,71E-03 | 2.2 (1.5-3.3) | 2,77E-03 |
| **rs4803221** | G/G | 19 (36.5) | 33 (63.5) | 3.9 (2.1-7.2) |  | 4.5 (2.2-9.4) |  |
|  | C/C | 244 (69.3) | 108 (30.7) | Baseline | | Baseline | |
|  | Carrier G | 197 (55.5) | 158 (44.5) | 1.8 (1.3-2.5) | 1,43E-01 | 2.5 (1.7-3.6) | 3,10E-03 |
|  | G/G | 182 (72.5) | 69 (27.5) | Baseline | | Baseline | |
|  | C/G | 222 (60.3) | 146 (39.7) | 1.7 (1.2-2.5) | 1,36E-03 | 2.4 (1.5-3.6) | 8,59E-05 |
| **rs4803222** | C/C | 37 (42.0) | 51 (58.0) | 3.6 (2.2-6.0) |  | 5.2 (2.8-9.7) |  |
|  | G/G | 182 (72.5) | 69 (27.5) | Baseline | | Baseline | |
|  | Carrier C | 259 (56.8) | 197 (43.2) | 2.0 (1.4-2.8) | 2,98E-02 | 2.8 (1.8-4.1) | 5,94E-04 |
|  | A/A | 106 (50.0) | 106 (50.0) | Baseline | | Baseline | |
|  | A/C | 229 (63.1) | 134 (36.9) | 0,6 (0.4-0.8) | 5,63E-08 | 0,5 (0.4-0.8) | 4,67E-07 |
| **rs8109886** | C/C | 106 (80.3) | 26 (19.7) | 0,3 (0.2-0.4) |  | 0,2 (0.1-0.4) |  |
|  | A/A | 106 (50.0) | 106 (50.0) | Baseline | | Baseline | |
|  | Carrier C | 335 (67.7) | 160 (32.3) | 0,5 (0.3-0.7) | 1,03E-05 | 0,4 (0.3-0.6) | 2,03E-05 |
|  | A/A | 182 (72.8) | 68 (27.2) | Baseline | | Baseline | |
|  | A/T | 223 (60.3) | 147 (39.7) | 1.8 (1.3-2.5) | 6,46E-07 | 2.4 (1.6-3.7) | 7,48E-08 |
| **rs8113007** | T/T | 36 (41.4) | 51 (58.6) | 3.8 (2.3-6.3) |  | 5.3 (2.8-9.8) |  |
|  | A/A | 182 (72.8) | 68 (27.2) | Baseline | | Baseline | |
|  | Carrier T | 259 (56.7) | 198 (43.3) | 2.0 (1.5-2.9) | 1,84E-05 | 2.8 (1.9-4.2) | 4,36E-07 |
|  | T/T | 264 (68.6) | 121 (31.4) | Baseline | | Baseline | |
|  | T/G | 163 (57.8) | 119 (42.2) | 1.6 (1.2-2.1) | 2,51E-05 | 0.3 (0.2-0.6) | 9,42E-06 |
| **rs8099917** | G/G | 14 (35.0) | 26 (65.0) | 4.1 (2.0-8.0) |  | 5.7 (2.5-12.9) |  |
|  | T/T | 264 (68.6) | 121 (21.4) | Baseline | | Baseline | |
|  | Carrier G | 177 (55.0) | 145 (45.0) | 1.8 (1.3-2.4) | 2,01E-04 | 0.5 (0.4-0.6) | 8,32E-08 |

*All SNPs were adjusted for demographic data: viral genotype, ethnicity, fibrosis, viral load and drug model. Results are shown as frequency; OR: odds ratio; CI: confidence interval.


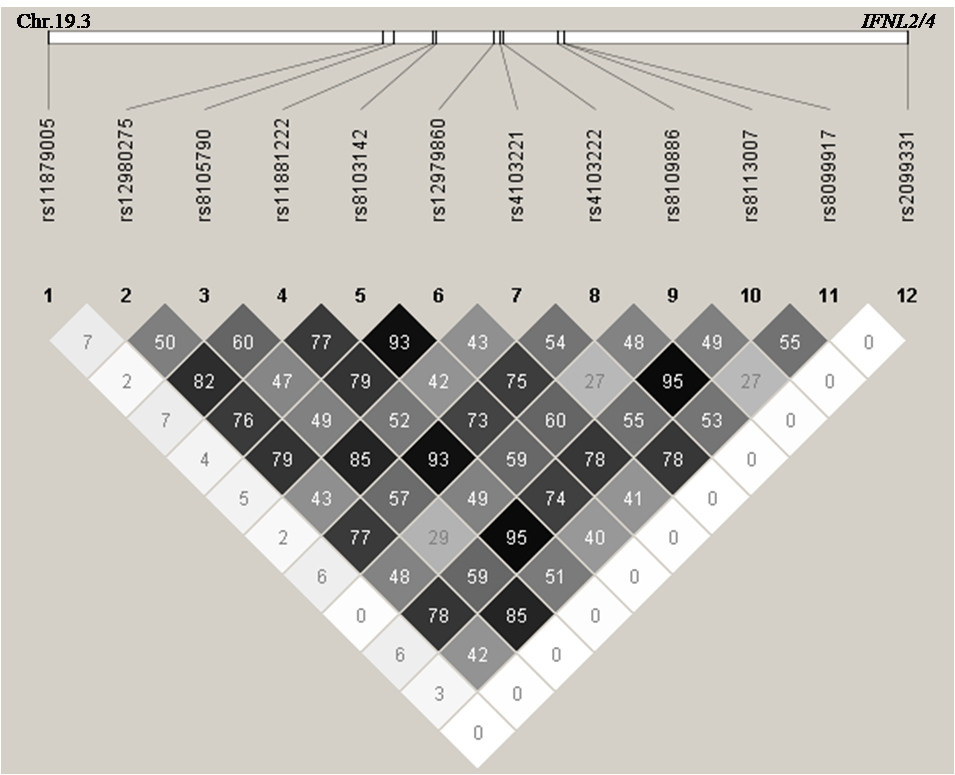


**Figure S1.** Pairwise linkage disequilibrium (LD) between SNPs of the *IFNL2/4* genes. The r^2^ values are proportional in grey shading, indicating the LD values between any two SNPs, and were defined by the method of confidence intervals implemented by the software Haploview.

**
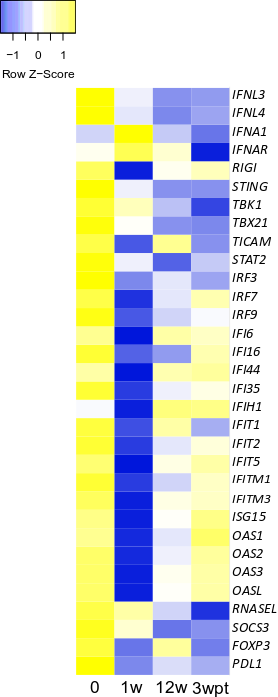
**

**Figure S2.** Heat map shown of the expression profile of the genes of the type I interferon pathway. Quantification of the expression of the genes of the IFN type I signaling pathway from whole blood in patients with HCC (n = 24) treated with Peg-IFNα / RBV, times 0, 1st, 12th week of treatment and 3rd week post-treatment. Representing the median of normalized values.

**
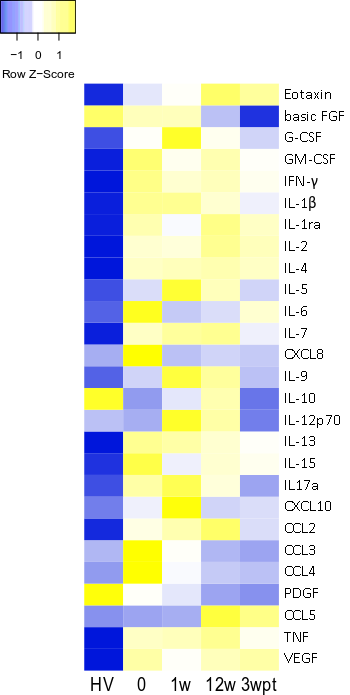
**

**Figure S3.** Heat map shown serum levels of the cytokines of the patients with CHC treated with Peg-IFNα/RBV and healthy volunteers. Quantification of cytokines Peg-IFNα / RBV treated patients at times 0, 72 hours, 1st, 3rd, 12th during treatment, and 3rd post-treatment (pt) week. Representing the median of the values obtained (patients n=24; healthy volunteer n=24).

**Figure S4***.* Serum levels of the IFN-α of the patients with CHC along with treatment with Peg-IFNα/RBV. On the x-axis, the time after the start of treatment in weeks and post-treatment is represented. Line graphic represents mean and standard deviation of IFN-α serum levels in pg/mL.


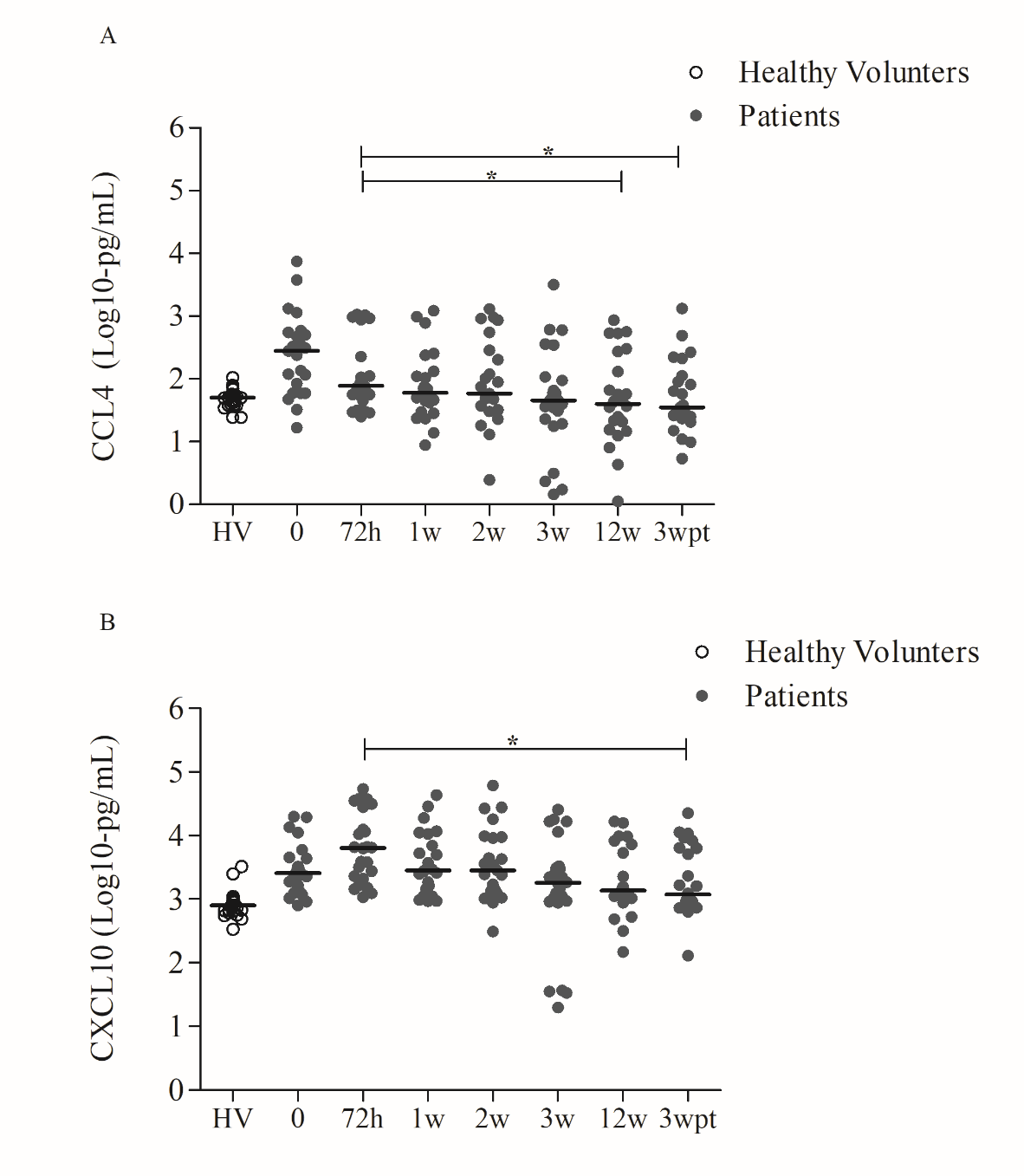


**Figure S5***.* Serum levels of the CCL4 and CXCL10 of the patients with CHC treated with Peg-IFNα/RBV and healthy volunteers who received one dose of Peg-IFNα. Quantification of untreated cytokines with Peg-IFNα / RBV patients at times 0, 72 hours, 1st, 3rd, 12th during treatment, and 3rd post-treatment (pt) week. (A) CCL4; (B) CXCL10. Differences between cytokine levels were assessed using the Mann Whitney

statistical test (* P <0.01, ** P <0.001, *** P <0.0001). Legends: HV=Healthy Volunteers.
